# The effectiveness of mindfulness-based cognitive therapy for children on anxiety, attentional control, and emotion regulation in children: a study protocol

**DOI:** 10.3389/fped.2025.1459434

**Published:** 2025-07-25

**Authors:** Habibeh Kholghi, Mojtaba Habibi Asgarabad, Khadijeh Abolmaali Alhosseini, Fahimeh Dehghani, Randye J. Semple

**Affiliations:** ^1^ Department of Counselling, Tehran North Branch, Islamic Azad University, Tehran, Iran; ^2^ Department of Psychology, Norwegian University of Science and Technology, Trondheim, Norway; ^3^ Department of Psychology, Faculty of Humanities and Social Sciences, Istinye University, Istanbul, Türkiye; ^4^ Department of Psychology, Tehran North Branch, Islamic Azad University, Tehran, Iran; ^5^ Department of Psychology and Educational Sciences, Yazd University, Yazd, Iran; ^6^ Department of Psychiatry and the Behavioral Sciences, Keck School of Medicine, University of Southern California, Los Angeles, CA, United States

**Keywords:** adolescents, anxiety, attentional control, children, cognitive therapy, emotion regulation, mindfulness, mindfulness-based cognitive therapy for children

## Abstract

**Introduction:**

Childhood stressors and adverse experiences can increase the likelihood of children developing mental disorders. The development of mindfulness-based cognitive therapy for children (MBCT-C) offers opportunities to improve emotional resiliency and reduce these children's vulnerabilities. The current study introduces the framework of a future clinical trial that seeks to examine the level of satisfaction and the efficacy of MBCT-C on anxiety, attentional control, and emotional regulation in children.

**Method:**

This research protocol outlines a quasi-experimental design that compares MBCT-C with an active control group. The intervention group will participate in a 12-session MBCT-C program, while the active control group will receive a 12-session program of life skills training (LST). Eighty children aged between 8 and 12 years will be randomly assigned to the intervention or the control group. Data using the Mindful Attention Awareness Scale for Children (MAAS-C), the Emotion Regulation Checklist (ERC), the State-Trait Anxiety Inventory for Children (STAI-CH), and the Attentional Control Scale for Children (ACS-C) will be collected at three times: baseline, post-intervention, and six months following the intervention. The Mindfulness Program Satisfaction Questionnaire (MPSQ) will assess participant satisfaction with the program. The effectiveness of MBCT-C will be evaluated using a conditional mixed regression model in STATA-18.

**Discussion:**

Providing a research protocol to conduct a clinical trial on MBCT-C before the intervention phase provides a precise evaluation of the goals and hypotheses, study method, assessment tools, and treatment outcomes.

## Introduction

Children frequently experience emotional problems, such as anxiety disorders (e.g., generalized anxiety disorder, separation anxiety), depression, obsessive-compulsive disorder, and disruptive mood regulation disorder in middle and late childhood (1, 2). Approximately 11.63% of children and adolescents worldwide meet the established criteria for a mental disorder (3), although it is essential to note that the prevalence of subclinical problems, particularly anxiety, is considerably greater (4). Mental problems experienced during childhood are often persistent and frequently continue into adulthood (5). Hence, it is imperative to consider the implementation of psychological interventions that enhance childhood resiliency and reduce the risk of subsequent internalizing and externalizing disorders (6, 7). Along with the contributing role of anxiety, emotion regulation (1, 8, 9) and attentional control (10) have been proposed as underlying factors in the psychopathology of children's emotional problems.

Anxiety is a future-oriented response to perceived threatening situations that are often characterized by ambiguity and uncertainty. Anxiety is a common, sometimes uncomfortable emotional experience. Only when it becomes severe is it considered a manifestation of psychological problems (11, 12). Chronic, uncontrollable anxiety can result in clinical distress and impaired performance (13, 14). Children often experience anxiety due to a wide range of transient or long-term stressors, which can be exacerbated by a limited capacity to effectively cope with such challenges (15, 16). When anxiety becomes severe, it impairs daily functioning, including social and school achievements (14, 17). For example, some children experience separation anxiety when they begin attending school, as they are separated from their parents and enter an unfamiliar environment (18). Others may feel socially anxious, fearing observation and negative judgements from others in social settings, which can lead to avoidance of many situations that require interpersonal interactions (19). Specific phobias also lead to avoidance of the source of fear and inability to tolerate the associated anxiety (20). Along with math (21) and test anxieties (22), which result in low grades and academic failure in children, these are common anxieties impair children's development.

Emotion regulation (ER) is an adaptive manner of managing emotions and feelings (8). ER capacity affects the interpretation of experiences, emotional expression, and responses to the emotions of others (23). Maladaptive coping strategies can disrupt emotion processing and aggravate emotional disturbances (8, 24, 25). Inadequate ER has received increasing attention as an underlying factor in childhood and adolescent mental disorders (26). Children who develop adaptive emotion regulation skills are more likely to have a better quality of life and are at lower risk for mental disorders (27). Strengthening ER skills is considered a preventive approach and a treatment goal to address emotional problems in children (26, 28). In one study, an ER-focused preventive intervention resulted in significant increases in emotional awareness, reductions in negative emotions, and reductions in internalizing problems in preschool children at risk for psychological problems (29). A recent meta-analysis found that a variety of psychosocial interventions were moderately effective in improving ER in children, adolescents, and transition-age youth (30).

Information processing in the human cognitive system takes place through the mechanism of attention control (AC). AC mechanisms facilitate the ability to direct and shift attention between stimuli and situations (31), selectively focusing on task-relevant information while ignoring irrelevant information (32). Difficulties managing attention to emotional and non-emotional stimuli have been associated with anxiety disorders and depression (33).

Cognitive processing deficits, especially the inability to shift attention from distressing thoughts, contribute to the persistence and exacerbation of worry and rumination (34, 35). Evidence supports the correlation of anxiety and depression with lower AC in children (36). The role of AC deficits is apparent in attention deficit hyperactivity disorder (ADHD) and some developmental problems (e.g., autism spectrum disorder) (37). A systematic review has provided evidence suggesting that enhancing attention capabilities contributes to a decrease in anxiety and depression (38).

The development of effective psychotherapies aimed at addressing emotional problems in children has made significant progress (14, 16, 39). Mindfulness-based interventions, in particular, are of growing interest. Mindfulness is an approach to life experiences that emphasizes attention to the present moment and with an attitude of non-judgment (40). Mindfulness, cultivated through meditation, involves bringing awareness to one's thoughts, emotions, and bodily sensations without engaging reactively to them (41, 42). Mindfulness-based interventions, which have been influential in evolving the third wave of cognitive-behavioral therapies, were developed initially for adults, but have now been adapted for children (43).

Mindfulness-based cognitive therapy (MBCT) was developed for relapse prevention in adult patients with recurrent depressive episodes (44, 45), and was later adapted for other problems and populations (46). One of these adaptations, MBCT-C, employs the same methodology as the adult program and shares analogous goals and objectives; however, it has been specifically tailored for children, which includes modifications to the duration of the sessions and the incorporation of child-friendly language and content (47). MBCT-C may be an effective intervention in mitigating emotional difficulties among children and adolescents (48, 49). A study conducted to investigate the effectiveness of MBCT-C on children and adolescents suffering from anxiety disorders who were at risk of bipolar disorder revealed that MBCT-C is effective in reducing clinical symptoms. Additionally, improvements in mindfulness were associated with improvements in anxiety and emotion regulation (50). The effectiveness of MBCT-C has been repeated in other clinical samples, including children with math anxieties (51), cancer (39), and internalizing disorders (52). The results of a meta-analysis, which compiled results from 3,977 child and adolescent mindfulness-based interventions, showed that the effect size of these interventions is small to moderate, and that the effectiveness appears to vary based on two factors: the type of facilitator and the characteristics of the program (53). Therefore, mindfulness-based interventions for children and adolescents in both clinical and non-clinical populations need further evaluation.

Children's clinical intervention programs should not be limited to clinical populations; their prophylactic effectiveness should also be examined in non-clinical populations. These programs may have significant benefits in preventing childhood problems (54). The primary aim of the present study protocol is to outline the theoretical framework and methodology of a quasi-experimental study to investigate the effectiveness of MBCT-C in managing non-clinical anxiety, emotion regulation, and attentional control in children. Five hypotheses are proposed:

Primary hypothesis

1. Following a 12-session MBCT-C program, children in this group will report significantly increased mindfulness compared to children attending a 12-session Life Skills Training (LST) program, as measured by the Mindful Attention Awareness Scale for Children (MAAS-C).

Secondary hypotheses

2. Following a 12-session MBCT-C program, children in this group will report the MBCT-C group will report significant decreases in state and trait anxiety as compared to children attending a 12-session LST program, as measured by the State-Trait Anxiety Inventory for Children (STAI-CH).
3. Following a 12-session MBCT-C program, children in this group will report a significant increase in emotion regulation as compared to children attending a 12-session LST program, as measured by the Emotion Regulation Checklist (ERC).
4. Following a 12-session MBCT-C program, participants in this cohort will report a significant increase in attentional control when compared to children participating in a 12-session LST program, as measured by the Attention Control Scale for Children (ACS-C).
5. The MBCT-C program will be acceptable to children and their parents as measured by recruitment, attendance, drop-out rates, and the Mindfulness Program Satisfaction Questionnaire (MPSQ).

## Method

### Study design

**Figure 1:**
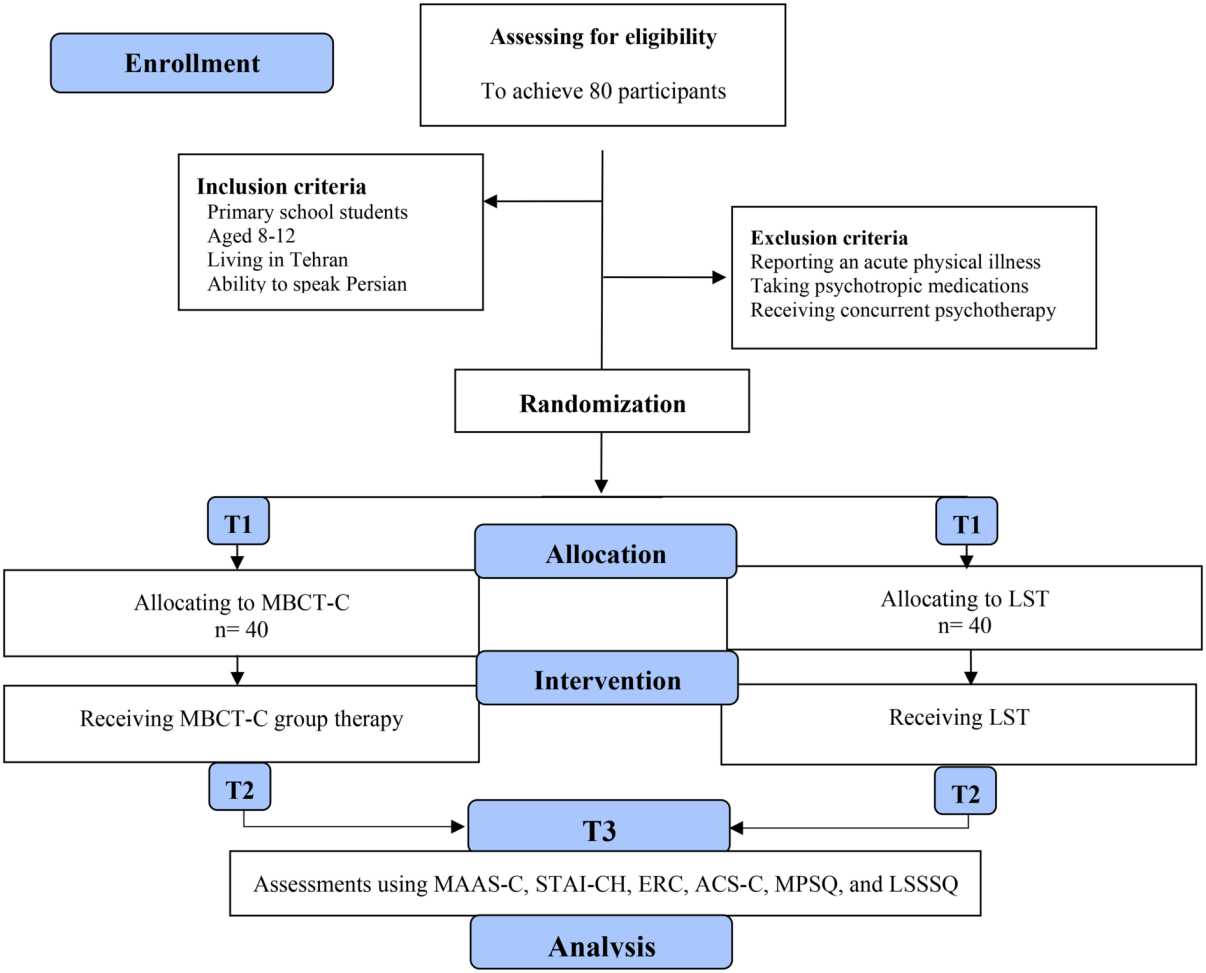
CONSORT diagram illustrating the study phases. T1: baseline; T2: Post-intervention; T3: 6-Month follow-up; MBCT-C: Mindfulness-based Cognitive Therapy for Children; MAAS-C-: Mindful Attention Awareness Scale- Adolescents; STAI-CH: State-Trait Anxiety Inventory for Children; ERC: Emotion regulation checklist; ACS-C: Attentional Control Scale for Children; MPSQ: Mindfulness Program Satisfaction Questioner (MPSQ).

This is a quasi-experimental study that comparing an MBCT-C program to an active control groupthe. Demographic data will be collected at baseline. Assessment data will be collected at baseline (T1), post-intervention (T2), and 6-month follow-up (T3) (Figure 1). Participants will be randomly assigned to either MBCT-C or LST using a software-generated random sequence. A stratified block randomization method will ensure balanced distribution by gender and age. A comparison of attendance and drop-out rates between the MBCT-C and LST groups will be one marker of program acceptability. Participants' adherence to the intervention will be evaluated via session attendance, group activity participation, and homework assignment compliance. To reduce potential bias, assessors will be blind to group allocation.

### Sample size

According to Thibault & Pedder (55) and Treves et al. (56), the required sample size for this study to achieve sufficient statistical power was determined using G*Power software (57). In this calculation, we used *a priori* power analysis with the following parameters: significance level *α* = .05; effect size = .5 (58); power (1-*β*).95; with two groups, corresponding to the study design. A minimum of 64 participants was estimated to detect hypothesized effects reliably. Recognizing the possibility of participant attrition, we incorporated a buffer to protect the power of the study. Specifically, we estimated a dropout rate of 20%, increasing the sample by an additional 14 participants (calculated as 20% of 64, rounded). This adjustment resulted in a total sample size of 77 (rounded to 80) participants. However, we acknowledge that this sample size limits our ability to detect smaller effect sizes. We have adjusted our expectations accordingly, focusing on larger effects as suggested by prior research (e.g., Goldberg et al. (59), for mindfulness interventions on stress.

### Recruitment and randomization procedures

In this study, our sampling framework encompasses four schools (two female-only and two male-only institutions) randomly selected from diverse districts across Tehran to ensure appropriate population representation. The target population comprises all middle school students in Tehran who, according to Iran's educational system, attend gender-segregated schools.

The necessary research permits will be obtained from Tehran's Education Department through the university's Research Vice-Chancellor's office. Group intervention sessions will be conducted after regular school hours in the counseling rooms of the participating schools, providing a quiet and appropriate environment for group therapeutic activities. This approach facilitates student access to the program while avoiding interference with their academic schedules.

Furthermore, school counselors will explain the research and introduce interested parents and students to the research team. Should these recruitment techniques be insufficient to obtain the desired sample size within a reasonable time, we will extend recruitment to additional schools within the Tehran education district.

Potential participants who contact the research assessment team will undergo an in-person screening to determine their eligibility. Inclusion criteria include: (a) primary school students, (b) age between 8 and 12 years old, (c) living in Tehran, and (d) ability to speak Persian. Exclusion criteria comprise (a) reporting an acute physical illness, (c) receiving psychotropic medications, and (e) receiving concurrent psychotherapy. Children who meet the criteria for this study and agree to participate will be enrolled with the consent of a parent or caregiver. Once the desired sample size is enrolled, participants will be randomized to the MBCT-C or LST group using online random allocation software (https://miniwebtool.com/de/number-randomizer/). The allocation will be made in a 1:1 ratio, ensuring equal representation of participants in each group.

### Procedure

Participants will be recruited from public elementary schools in Tehran. We will employ a staged recruitment approach, enrolling four cohorts of 20 children each (80 total). As each cohort of 20 children is recruited, they will be randomly assigned to either intervention or control groups (10 per group), and the 12-week programs will begin. While the first cohort undergoes treatment, recruitment for subsequent cohorts will continue.

Interested children will participate in a brief eligibility screening. Eligible students and their parents will receive comprehensive information about the study. Participation requires written informed consent from a parent or guardian and verbal assent from the child. Participants assigned to the intervention group will attend weekly 90-minute MBCT-C sessions for 12 weeks. The control group will participate in a 12-week LST program. Each program will be facilitated by separate instructors trained and invested in their respective models to prevent potential bias.

Protocol adherence will be monitored using the MBCT-C adherence scale and an equivalent measure for LST. A trained supervisor will review recorded sessions and provide feedback to facilitators to ensure standardized protocol implementation. Participants will complete the T2 assessment battery at the end of the intervention and the T3 follow-up assessment six months later. The Client Satisfaction Questionnaire (CSQ-8) will measure participant satisfaction with both programs.

### Mindfulness-based cognitive-behavioral therapy-children (MBCT-C)

MBCT-C (47) is a manualized 12-week treatment protocol that focuses on addressing attentional problems and anxiety-spectrum disorders in children. This protocol is designed for children between 8 and 12 years old and includes a variety of meditation and mindfulness techniques to improve cognitive, emotional, and physical awareness.

MBCT-C consists of three sequential steps to improve mindfulness. In the first phase (Sessions 1–3), in addition to preparing participants to overcome the obstacles ahead and how to do the tasks, exercises for conscious breathing, body awareness, and mindfulness will be presented. The second step of MBCT-C (sessions 4–10), which constitutes the principal portion of interventions, is aimed at thoughts, emotional experiences, and physical sensations in a more adept manner ways. Participants will engage in exercises designed to enhance mindfulness in each of their five senses. Additionally, based on informed and non-judgmental observations, they will examine various alternative ways of responding to challenging thoughts, emotions, and external events. In the final stage of the program (sessions 11 and 12), participants are taught to integrate mindfulness practices into their daily routines. Participants will discuss maintaining a daily practice of mindfulness and discuss benefits that may have been experienced during the program. Three months after the end of the program, each participant will receive a letter written by themselves in session 11 to motivate themselves to continue a consistent daily practice. At each session, written session summaries, homework assignments, poems, or stories related to that session, are provided to the participants (Table 1).

**Table 1:** The session-to-session content of the MBCT-C protocol [Semple and Lee, (47)].

Sessions	Content of session	Assignments
Session 1 Being on automatic pilot	• The main concepts and structure of sessions. • Differences between habitual life and mindful life.	• Awareness of thoughts and emotions • Awareness of automatic behaviors • Mindful breathing
Session 2 Being Mindful	• Paying attention to the present moment. • Increasing the duration of being mindful. • Dealing with internal obstacles (including thoughts and emotions). • Differences in reactions when awareness is incomplete compared to when there is a mindful state.	• Practicing breathing exercises and mindful eating meditation throughout the week
Session 3 Who Am I?	• Considering thoughts as possibilities rather than facts. • Being aware of the presence of thoughts. • Improving reactions with mindfulness.	• Mindful breathing • Mindfulness of the body
Session 4 A Taste of Mindfulness	• Considering other aspects of the present experience. • Find new ways to experience. • Exploring more awareness, compassion, and capacity for challenging situations.	• Three-minute breathing space • Mindful yoga movements • Tasting fruits
Session 5 Music to Our Ears	• Engaging in attentive listening to voices and sounds. • Identifying psychological obstacles that hinder the ability to maintain attention.	• Three-minute breathing space • Mindfulness of the body • Mindful listening
Session 6 Sound Expressions	• Exploring the role of thoughts and feelings on vocal expression. • Increasing awareness of finding more choices in response to experiences.	• Three-minute breathing space • Mindfulness of the body • Unpleasant sounds
Session 7 Practice Looking	• Non-judgmental attention to what we see. • Mindful observation exercises.	• Three-minute breathing space • Seeing the little details
Session 8 Strengthening the Muscle of Attention	• Developing awareness of both external and internal perceptions. • Disabling the autopilot mode and cultivating a state of mindful awareness. • Avoiding blame and cultivating compassion during meditations.	• Three-minute breathing space • Choosing to be aware • Seeing five new things
Session 9 Touching the World	• Bringing awareness to everyday experience. • Taking notes about experiences.	• Three-minute breathing space • Mindfulness of the body • Mindful Touching
Session 10 What the Nose Knows	• Practicing mindful smelling. • Determining how to react to unpredictable and uncontrollable events.	• Three-minute breathing space • Mindful Yoga movements • Mindful smelling
Session 11 Life Is Not Rehearsal	• Accepting the presence of judgmental thoughts without engaging. • Cultivating acceptance and expanding greater compassion.	• Three-minute breathing space • Letter to myself
Session 12 Living with Presence, Compassion, and Awareness	• Involved in practical strategies for building a daily routine based on mindfulness.	• Letter from therapist to child • Daily practice calendar

### Life skills training (LST)

LST is a comprehensive 12-week group program, developed by GJ Botvin and KW Griffin (60) developed to help participants practice ten essential life skills. The skills covered include self-awareness, empathy, interpersonal communication, coping with stress, decision-making, problem-solving, emotional management, creative thinking, critical thinking, and effective communication. Like the MBCT-C program, LST incorporates practice during sessions and assigns homework. Based on the sessions' practices, upon learning each skill, participants reflect on a recent challenging situation in their lives and utilize the skills acquired to reevaluate that situation, developing appropriate responses. During each session, a facilator elucidates the skills to the participants and facilitate their learning through constructive feedback. As part of their homework, participants are encouraged to effectively implement the skills they have acquired to manage various situations encountered throughout the week. In each session, the homework from the previous session is collectively reviewed in a shared space (Table 2). Two distinct therapists will facilitate the LST and MBCT-C group sessions. We have selected therapists with specialized training and experience in their respective programs. This approach mitigates the risk of bias if a single individual were to conduct both programs, particularly given varying levels of expertise or personal preference.

**Table 2:** Introduction to the life skills training program.

Sessions	Main contents	Assignments
Session 1 Introduction	• Warming up • Introduction to the program • Fostering Engagement and Mutual Respect • Guiding participants on doing homework exercises	• None
Session 2 Self-Awareness	• Promoting self-reflection on values, beliefs, and traits • Recognizing personal strength points • Identifying personal weaknesses • Expressing their emotions • Encouraging self-reflection, self-acceptance, and personal growth	• Self-monitoring
Session 3 Empathy	• Identify and effectively address the emotions of people • Practicing active listening, non-judgmental language	• Documenting their experiences with others
Session 4 Interpersonal Communication	• Active listening • Clear expression of ideas • Respectful dialoguing	• Active Listening Exercise • Perspective-Taking Exercise
Session 5 Effective Relationship	• Creating meaningful connections • Doing joint activities • Attention to the interests of friends and relatives • Resolve conflicts constructively • Strengthening mutual understanding	• Monitoring Previous skills • Practicing improving current relationships
Session 6 Coping Stress	• Breathing exercises • Adopting healthier habits • Engaging in physical activity	• Breathing exercises • Building good habits • Doing exercise
Session 7 Problem Solving	• Identifying the problem • Brainstorming • Evaluation of the solutions • Implementing the most optimal one • Evaluating the consequences	• Practicing on daily problems and documenting them
Session 8 Decision-Making	• Evaluating costs and benefits • Searching for Information and Insights • Collaborative decision-making processes • Mitigating risks and uncertainties • Confident and responsible decision-making	• Documenting decision-making for daily situations
Session 9 Creative Thinking	• Cultivating an innovative mindset • Exploring Unconventional Solutions • Embracing imagination and divergent thinking • Overcoming mental blocks and biases	• Brainstorming, mind mapping, and lateral thinking regarding an interesting issue
Session 10 Critical Thinking	• Logical Inquiry • Evaluating Information with Objectivity • Identifying Assumptions and Biases • Drawing Well-Reasoned Conclusions	• Evaluating the source's credibility, bias, and potential motives in a newspaper
Session 11 Emotional Management	• Implementing progressive muscle relaxation • Practicing cognitive reframing • Identifying emotional triggers • Substituting avoidance with experiencing.	• Identifying emotional triggers and using a strategy to expose them effectively
Session 12 Maintain achievements	• Reviewing and discussing the previous sessions • Implementing the skills in everyday life • Continuing monitoring • Documenting worksheets and diaries	• None

### Protocol adherance

The study supervisor monitors protocol adherence through multiple methods. First, they review video recordings of all sessions. Second, they hold supervision meetings with the therapist every two weeks. Third, they complete standardized fidelity assessments after each session.

For MBCT-C sessions, the supervisor uses the validated MBCT-C Adherence Scale. This tool measures how closely the therapist follows the established protocol. For LST sessions, the supervisor uses the Life Skills Training Fidelity Monitoring Checklist developed by Botvin and colleagues. Both assessment tools evaluate content delivery, participant engagement, and implementation of core techniques.

The supervisor provides feedback to facilitators immediately after reviewing each session. This allows facilitators to address any protocol deviations quickly. Such close monitoring ensures consistent program delivery across all four cohorts of participants.

### Measures

#### Mindful attention awareness scale**-**children (MAAS-C)

This unpublished self-report tool consists of 15 items to measure mindfulness in children (61). Participants will respond to the questions using a Likert scale ranging from 1 (*almost never*) to 6 (*almost always*). All 15 items are phrased negatively and reversely scored. This scoring approach means individuals receiving elevated scores demonstrate stronger mindfulness capabilities. The original MAAS-C has a good internal consistency among children (*α* = .84) (62).

#### State-trait anxiety inventory for children (STAI-Ch)

The STAI-CH is a self-report measure of anxiety levels in children (63). Two independent scales of this inventory are state anxiety (S anxiety) and trait anxiety (T anxiety). S anxiety assesses anxiety as a temporary emotional condition, and T anxiety evaluates anxiety as a trait of personality. Both scales consist of 20 items, each rated on a 3-point Likert scale from 1 (*rarely*) to 3 (*often*). The overall score for each variable ranges from 20 to 60. The State-Trait Anxiety Inventory for Children (STAI-CH) has exhibited strong psychometric properties. The internal consistency coefficients for both scales surpassed.80 (63). In addition, it has been shown that the psychometric properties of the Persian version of STAI-CH have good levels of internal consistency (*α* = .89) (64).

#### Emotion regulation checklist (ERC)

ERC is a commonly employed other-report (e.g., completed by parents or teachers) utilized to evaluate the emotion regulation capacities of children and adolescents (65). ERC includes 24 items scored on a 4-point Likert scale, from 1 (*never*) to 4 (*almost always*). Items are categorized into two subscales, Lability/Negativity and Emotion Regulation. Lability/Negativity evaluates how individuals encounter negative emotions and exhibit emotional instability. Emotion Regulation evaluates the capacity to regulate emotional reactions and engage in self-soothing actions. Numerous research investigations showed the ERC's robust psychometric characteristics. The internal consistency of ERC has been determined to be satisfactory for the Lability/Negativity (*α* = .96) and the Emotion Regulation subscale (*α* = .83). The ERC exhibits strong convergent validity when compared to other assessments of behavioral problems and social skills (66). The capacity to detect changes has also been evidenced in intervention studies, wherein enhancements in the aptitude to regulate emotions were correlated with a reduction in negative emotional states and an elevation in positive emotional states. The present study will use the Persian version of the ERC. This checklist has high internal consistency (*α* = .90) in an Iranian population and has convergent validity and acceptable discriminant validity (67).

#### Attentional control scale for children (ACS-C)

ACS-C is a 20-item self-report scale (68) to assessing attentional control among children. Each item is rated on a five-point Likert scale that ranges from 1 (*almost never*) to 4 (*always*). Items evaluate three distinct aspects of attentional control: focusing, shifting, and flexibility. The internal consistency of ACS-C has been reported to be good in different studies [*α* = .78 and .74 (69, 70)]. The present research will utilize the Persian adaptation of the ACS-C. As this version has not yet been standardized for use in Iran, the internal consistency of the questionnaire will be evaluated during the study's second phase.

### Satisfaction and adherence measures

#### Mindfulness program satisfaction questionnaire (MPSQ)

MPSQ was developed by Semple (2016) using the items of the original version of the Client Satisfaction Questionnaire [CSQ (71)]. It includes 8 items rated on a 4-point Likert scale. Half of the items are scored directly (including 5, 4, 2, and 8), and the other half is scored in reverse (1, 3, 6, and 7). Scores range from 8 to 32, and higher scores indicate higher satisfaction with the MBCT-C program.

#### Life skills training satisfaction questionnaire (LSTSQ)

We will use an adapted version of the Client Satisfaction Questionnaire [CSQ-8 (71)] to measure participant satisfaction in the LST group. This adapted questionnaire maintains the original 8-item structure with a 4-point Likert scale, with modifications to reflect LST-specific content. Scores range from 8 to 32, with higher scores indicating greater satisfaction with the LST program.

### Ethical standards

We registered the present study in the Iranian Registry of Clinical Trials (IRCT; IRCT20230922059488N1). We will follow the ethical guidelines related to human experiments. In addition, we will adhere to the principles outlined in the Declaration of Helsinki in 1975, which was subsequently revised in 2008, in all study phases. Given that the research will be conducted with children under age 18, a formal informed consent document must be signed by a guardian or parent of everyone included in the study. Children will provide verbal assent to participate. Participants may discontinue the intervention at any point during the research voluntarily.

Participant anonymity will be maintained through the assignment of unique identification codes in place of personal information. Names and contact details will be replaced with pseudonyms during data collection. Each participant will receive a distinct identifier that will prevent direct linkage to individual identities. The coding key will be stored separately from the main dataset in a secure location. Data storage will follow standard security protocols. An encrypted database will house all collected information, with access restricted to designated research team members.

The research director, ethics committee, and IRCT will be duly informed of any probable modifications made to the study, and these changes will be subsequently documented in the IRCT. This study protocol received approval from the local ethics council at the Azad University- North Tehran Branch (ethics code: IR.IAU.TNB.REC.1402.067).

### Statistical strategy

The study results will adhere to the guidelines established by the SPIRIT guidelines (72). The analysis will be conducted using the STATA-18 software. After data collection and curation, to determine data consistency, we assess the status of outliers by calculating both the average and a 5% reduced average (*p* < .05). We shall also analyze demographic differences in the baseline utilizing independent *t*-tests and chi-square tests. Furthermore, an intention-to-treat (ITT) analysis will be conducted should any participant miss more than three intervention sessions. This approach ensures that missing data does not significantly bias the study's primary analysis, remaining below 5% of the total data. In such instances, multiple imputations will replace missing values with alternative ones.

To evaluate the study outcomes, we will use a conditional mixed regression model to simultaneously compare the fixed and random effects of two groups (MBCT-C vs. LST) at the three assessment times (T1, T2, T3). The fixed effects will include group, time, and the group-by-time interaction, enabling the assessment of changes in the main outcomes across time and between groups. Participant ID will be treated as a random effect to account for repeated measurements within individuals. Where appropriate, baseline variables such as age and gender will be included as additional fixed effects to examine their potential role in moderating intervention outcomes. All analyses in the present study will evaluate the results at a significant level (*p* < .05). The Reliable Change Index (73) will be applied to examine clinically significant changes in primary and secondary outcomes before and after MBCT-C. A statistically significant difference will be represented by an RCI greater than 1.96.

## Result

This research is a part of the doctoral thesis of the first author of this article, whose proposal was approved in February 2023 at North Tehran Azad University. The ethical code of this study was presented by the ethics committee of the corresponding university on September 1, 2023. The initial screening of participants is currently underway. The seating required for this intervention and the prerequisites for the sessions are fully provided. When sampling and screening of participants is finished, the interventions will begin.

## Discussion

Recent findings regarding doubts about the effectiveness of mindfulness interventions for adolescents have raised concerns about the efficacy of these interventions for younger individuals (53, 74). This study protocol focuses on a younger age group, specifically children aged 8–12. According to this issue, the research explores whether early intervention can enhance mindfulness, reduce anxiety, improve emotional regulation, and boost attention control. The significance of these characteristics is well-established in a diverse array of childhood emotional and behavioral disorders. If MBCT-C can address these problems efficiently, the participants will likely experience positive outcomes.

In this study, it is assumed that anxiety is a prevalent issue among children, demanding intervention when it develops into a functionally impairing disorder. Untreated anxiety issues in children increase their susceptibility to future occurrences of depression, substance abuse, and further emotional difficulties (75–77). The MBCT-C program in this study provides activities and skills training designed to enable children to manage anxiety and overcome emotional avoidance more effectively. Children who include these meditation techniques in their daily routines may feel enhanced psychological well-being, resulting in reduced psychological distress when confronted with anxiety-inducing situations. Moreover, they may be more inclined to face anxiety-provoking situations rather than avoiding them (78).

The absence of proficient emotion regulation skills often results in exacerbated difficulties when attempting to cope with anxiety and stress (79). Children who rely on avoidance or maladaptive coping strategies, such as excessive worry, are at an increased risk of developing anxiety disorders and depression (80, 81). Enhancing emotion regulation skills, especially improved awareness, non-judgmental acceptance, and cognitive reappraisal, is a potential benefit of MBCT-C. Consequently, providing children with these strategies can effectively equip them to cope with emotional challenges. Children who employ adaptive emotion regulation strategies to cope with anxiety can decrease their susceptibility to internalizing and externalizing disorders (82). The development and maturation of adolescents is also facilitated by the cultivation of emotion regulation skills in children (83).

MBCT-C utilizes mindfulness to improve attentional control through sensory-focused activities with children during sessions (taste, hearing, seeing, touch, and smelling), which increases ongoing awareness and attention to non-distracting sensations (84). This study protocol aims to evaluate MBCT-C's efficacy and satisfaction. Children need to develop attentional control, which facilitates the regulation of related emotions/thoughts/behaviors and may improve social-emotional and academic performance (85). This study protocol establishes a controlled framework to assess the efficacy of MBCT-C in children, which will enhance our ability to debate and make conclusions on the effect size and the generalizability of the findings within Iranian children. The study design exhibits two significant limitations requiring careful consideration when interpreting and extrapolating the findings. First and foremost, due to constraints in both financial and human resources, it was unfeasible to carry out the study with a larger sample size. Furthermore, given that the study's sampling is conducted within educational institutions, the potential exists for transmitting session-related information from the intervention group to the control group. If the findings of the present study demonstrate substantial efficacy in these interventions, further research should investigate their effectiveness and participant satisfaction across diverse samples of Iranian children. Additionally, it is advised that future research endeavors encompass a comparative analysis of the efficacy of the treatment program relative to other established treatment programs.

Future research should prioritize larger-scale studies if this study demonstrates MBCT-C's efficacy. Longitudinal studies are also needed to investigate the sustained effects of MBCT-C on children's mental health and academic outcomes over time. Similarly, future comparative studies between MBCT-C and other well-established treatments (e.g., cognitive-behavioral therapy or other mindfulness-based programs) could provide a more comprehensive understanding of its efficacy and cost-effectiveness. Examining participant satisfaction and program feasibility in different Iranian settings, including rural or underserved areas, would provide additional empirical support for the scalability of MBCT-C across diverse populations. Moreover, qualitative methods (e.g., child and parent interviews) would also provide further interpretation of the intervention's acceptability and mechanisms of change.
